# Combined Effect of Buoyancy Force and Navier Slip on MHD Flow of a Nanofluid over a Convectively Heated Vertical Porous Plate

**DOI:** 10.1155/2013/725643

**Published:** 2013-10-03

**Authors:** Winifred Nduku Mutuku-Njane, Oluwole Daniel Makinde

**Affiliations:** ^1^Mechanical Engineering Department, Cape Peninsula University of Technology, P.O. Box 1906, Bellville 7635, South Africa; ^2^Faculty of Military Science, Stellenbosch University, Private Bag X2, Saldanha 7395, South Africa

## Abstract

We examine the effect of magnetic field on boundary layer flow of an incompressible electrically conducting water-based nanofluids past a convectively heated vertical porous plate with Navier slip boundary condition. A suitable similarity transformation is employed to reduce the governing partial differential equations into nonlinear ordinary differential equations, which are solved numerically by employing fourth-order Runge-Kutta with a shooting technique. Three different water-based nanofluids containing copper (Cu), aluminium oxide (Al_2_O_3_), and titanium dioxide (TiO_2_) are taken into consideration. Graphical results are presented and discussed quantitatively with respect to the influence of pertinent parameters, such as solid volume fraction of nanoparticles (*φ*), magnetic field parameter (Ha), buoyancy effect (Gr), Eckert number (Ec), suction/injection parameter (*f*_*w*_), Biot number (Bi), and slip parameter (**β**), on the dimensionless velocity, temperature, skin friction coefficient, and heat transfer rate.

## 1. Introduction

Magnetohydrodynamic (MHD) boundary layer flow of an electrically conducting viscous incompressible fluid with a convective surface boundary condition is frequently encountered in many industrial and technological applications such as extrusion of plastics in the manufacture of Rayon and Nylon, the cooling of reactors, purification of crude oil, textile industry, polymer technology, and metallurgy. As a result, the simultaneous occurrence of buoyancy and magnetic field forces on fluid flow has been investigated by many researchers [1–5]. In their investigations, all the authors mentioned above assumed the no-slip boundary conditions. However, more recently, researchers have investigated the flow problem taking slip flow condition at the boundary [6–9]. 

On the other hand, with the advent of nanofluids, there has been wide usage of recently discovered smart fluid in many industrial and biomedical applications. Nanofluid concept is employed to designate a fluid in which nanometer-sized particles are suspended in conventional heat transfer base fluids to improve their thermal physical properties. Nanoparticles are made from various materials, such as metals (Cu, Ag, Au, Al, and Fe), oxide ceramics (Al_2_O_3_, CuO, and TiO_2_), nitride ceramics (AlN, SiN), carbide ceramics (SiC, tiC), semiconductors, carbon nanotubes, and composite materials such as alloyed nanoparticles or nanoparticle core-polymer shell composites. It is well known that conventional heat transfer fluids, such as oil, water, and ethylene glycol, in general, have poor heat transfer properties compared to those of most solids. Nanofluids have enhanced thermophysical properties such as thermal conductivity, thermal diffusivity, viscosity, and convective heat transfer coefficients compared with those of base fluids like oil or water [10]. Several authors [11–14] have conducted theoretical and experimental investigations to demonstrate that nanofluids distinctly exhibit enhanced heat transfer properties which goes up with increasing volumetric fraction of nanoparticles. Further studies on nanofluids have been currently undertaken by scientists and engineers due to their diverse technical and biomedical applications such as nanofluid coolant: electronics cooling, vehicle cooling, transformer cooling, computers cooling, and electronic devices cooling; medical applications: magnetic drug targeting, cancer therapy, and safer surgery by cooling; process industries; and materials and chemicals: detergency, food and drink, oil and gas, paper and printing, and textiles. 

According to Aziz [15], the concept of no-slip condition at the boundary layer is no longer valid for fluid flows in microelectromechanical systems and must be replaced by slip condition. The slip flow model states a proportional relationship between the tangential components of the fluid velocity at the solid surface to the shear stress on the fluid-solid interface [16]. The proportionality is called the slip length, which describes the slipperiness of the surface [7]. Many researchers studied the effect of linear momentum and nonlinear slip on the MHD boundary layer flow with heat/mass transfer of free/forced/combined convection past different geometries [17–20]. In spite of the importance of MHD related studies on boundary layer flow problems, the possibility of fluid exhibiting apparent slip phenomenon on the solid surface has received little attention. 

The aim of the present study is to investigate the combined effects of buoyancy, magnetic field, suction, Navier slip, and convective heating on a steady boundary layer flow over a flat surface. In the subsequent sections the boundary layer partial differential equations first transformed into a system of nonlinear ordinary differential equations before being solved numerically using a shooting method together with the fourth-order Runge-Kutta-Fehlberg integration scheme. A graphical representation of the pertinent parameters on the flow field and heat transfer characteristics is displayed and thoroughly discussed. To our best of knowledge, the investigations of the proposed problem are new, and the results have not been published before.

## 2. Model Formulation 

**Figure 1:**
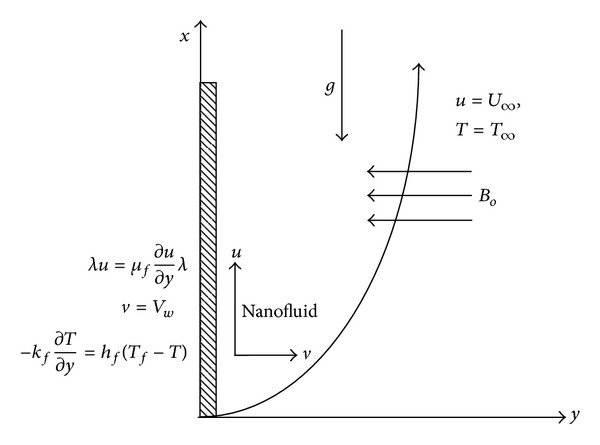
Flow configuration and coordinate system.

The steady laminar incompressible two-dimensional MHD boundary layer flow of an electrically conducting water-based nanofluid past a convectively heated porous vertical semiinfinite flat plate under the combined effects of buoyancy forces and Navier slip is considered. The nanofluids contain three different types of nanoparticles: Cu, Al_2_O_3_, and TiO_2_. Let the *x*-axis be taken along the direction of plate, and let *y*-axis be normal to it. The left side of the plate is assumed to be heated by convection from a hot fluid at temperature *T*_*f*_, which provides a heat transfer coefficient *h*_*f*_, while the right surface is subjected to a stream of an electrically conducting cold nanofluid at temperature *T*_*∞*_ in the presence of a transverse magnetic field of strength *B*_0_ applied parallel to the *y*-axis, as shown in Figure 1. The induced magnetic field due to the motion of the electrically conducting fluid is negligible. It is also assumed that the external electrical field is zero and that the electric field due to the polarization of charges is negligible (see Table 1).

Assuming a Boussinesq incompressible fluid model, the continuity, momentum, and energy equations describing the flow can be written as

(1) ∂u∂x+∂v∂y=0,u∂u∂x+v∂u∂y=U∞dU∞dx+μnfρnf∂2u∂y2+βnfg(T−T∞)−σnfB02(u−U∞)ρnf,u∂T∂x+v∂T∂y=knf(ρcp)nf∂2T∂y2+μnf(ρcp)nf(∂u∂y)2+σnfB02(ρcp)nf(u−U∞)2.

The boundary conditions at the plate surface and at the free stream may be written as

(2) $$\begin{array}{c} \lambda u\left(x,0\right)=\mu_{f}\frac{\partial u}{\partial y}\left(x,0\right),\;\;v\left(x,0\right)=V_{w},\\ -k_{f}\frac{\partial T}{\partial y}\left(x,0\right)=h_{f}\left[T_{f}-T\left(x,0\right)\right],\\ u\left(x,\infty \right)=U_{\infty }\left(x\right),\;\;T\left(x,\infty \right)=T_{\infty }, \end{array}$$

where (*u*, *v*) are the velocity components of the nanofluid in the *x*- and *y*-directions, respectively, *T* is the nanofluid temperature, *U*_*∞*_(*x*) = *ax* is the free stream velocity (which implies that the free stream fluid velocity is increasing with axial distance along the plate surface), *T*_*∞*_ is the free stream temperature, *g* is acceleration due to gravity, *λ* is the slip coefficient, *μ*_*nf*_ is dynamic viscosity of the nanofluid, *ρ*_*nf*_ is density of the nanofluid, *k*_*nf*_ is thermal conductivity of the nanofluid, *σ*_*nf*_ is electrical conductivity of the nanofluid, (*ρc*_*p*_)_*nf*_ is heat capacity at constant pressure of the nanofluid, and *β*_*nf*_ is volumetric expansion coefficient of the nanofluid which are defined as [21, 22]

(3) $$\begin{array}{c} \mu_{nf}=\frac{\mu_{f}}{{\left(1-\varphi \right)}^{2.5}},\;\;\rho_{nf}=\left(1-\varphi \right)\rho_{f}+\varphi \rho_{s},\\ \beta_{nf}=\left(1-\varphi \right)\beta_{f}+\varphi \beta_{s},\;\;\alpha_{nf}=\frac{k_{nf}}{{\left(\rho c_{p}\right)}_{nf}},\\ \frac{k_{nf}}{k_{f}}=\frac{\left(k_{s}+2k_{f}\right)-2\varphi \left(k_{f}-k_{s}\right)}{\left(k_{s}+2k_{f}\right)+\varphi \left(k_{f}-k_{s}\right)},\\ {\left(\rho c_{p}\right)}_{nf}=\left(1-\varphi \right){\left(\rho c_{p}\right)}_{f}+\varphi {\left(\rho c_{p}\right)}_{s},\\ \sigma_{nf}=\left(1-\varphi \right)\sigma_{f}+\varphi \sigma_{s}, \end{array}$$

where *φ* is the nanoparticle volume fraction (*φ* = 0 correspond to a regular fluid), *ρ*_*f*_ and *ρ*_*s*_ are the densities of the base fluid and the nanoparticle, respectively, *β*_*f*_ and *β*_*s*_ are the thermal expansion coefficients of the base fluid and the nanoparticle, respectively, *k*_*f*_ and *k*_*s*_ are the thermal conductivities of the base fluid and the nanoparticles, respectively, (*ρc*_*p*_)_*f*_ and (*ρc*_*p*_)_*s*_ are the heat capacitance of the base fluid and the nanoparticle, respectively, and *σ*_*s*_ and *σ*_*f*_ are the electrical conductivities of the base fluid and the nanofluid, respectively.

In order to simplify the mathematical analysis of the problem, we introduce the following dimensionless variables:

(4) $$\begin{array}{c} \eta ={\left(\frac{a}{\upsilon_{f}}\right)}^{1/2}y,\;\;\psi ={\left(a\upsilon_{f}\right)}^{1/2}xf\left(\eta \right),\\ \theta \left(\eta \right)=\frac{T-T_{\infty }}{T_{f}-T_{\infty }}, \end{array}$$

where *η* is the similarity variable and *ψ* is the stream function defined as

(5) $$\begin{array}{c} u=\frac{\partial \psi }{\partial y},\;\;v=-\frac{\partial \psi }{\partial x}. \end{array}$$

After introducing (5) into (1) and (2), we obtain the following ordinary differential equations:

(6) f′′′+(1−φ)2.5(1−φ+φρsρf)ff′′  −(1−φ)2.5(1−φ+φρsρf)(f′)2  +(1−φ)2.5(1−φ+φρsρf)  +Gr(1−φ)2.5(1−φ+φρsρf)(1−φ+φβsβf)θ  −Ha(1−φ)2.5(1−φ+φσsσf)(f′−1)=0,θ′′+Pr kf[1−φ+φ(ρcp)s/(ρcp)f]knffθ′  +Pr Ec kfknf(1−φ)2.5(f′′)2+Ha Pr Ec kfknf  ×(1−φ+φσsσf)(f′−1)2=0.

Taking into account the variable plate surface permeability and the hydrodynamic slip boundary functions defined, respectively, as

(7) $$\begin{array}{c} V_{w}=-f_{w}{\left(a\upsilon_{f}\right)}^{1/2},\;\;\lambda u\left(x,0\right)=\mu_{f}\frac{\partial u}{\partial y}\left(x,0\right), \end{array}$$

the boundary conditions are

(8) $$\begin{array}{c} f\left(0\right)=f_{w},\;\;f^{\prime }\left(0\right)=\beta f^{\prime \prime }\left(0\right),\\ \theta^{\prime }\left(0\right)=\text{Bi}\left[\theta \left(0\right)-1\right],\\ f^{\prime }\left(\infty \right)=1,\;\;\theta \left(\infty \right)=0, \end{array}$$

where a prime symbol denotes derivative with respect to *η*, *f*_*w*_ is a constant with *f*_*w*_ > 0 representing suction rate at the plate surface, *f*_*w*_ < 0 corresponds to injection, *f*_*w*_ = 0 shows an impermeable surface, *λ* = 0 represents highly lubricated surface, and *λ* = *∞* corresponds to a normal surface. The local Reynolds number (*Re*_*x*_), Grashof number (Gr), Hartmann number (Ha), Prandtl number (Pr), Eckert number (Ec), slip parameter (*β*), and Biot number (Bi), are defined as

(9) Rex=U∞xυf,  Gr=βfg(Tf−T∞)U∞a,Ha=σfBo2ρfa,  Pr=υfαf,Ec=U∞2Cpf(Tf−T∞),  β=μfλaυf,Bi=hfkfυfa.

The physical quantities of practical significance in this work are the skin friction coefficient *C*_*f*_ and the local Nusselt number Nu, which are expressed as

(10) $$\begin{array}{c} C_{f}=\frac{\tau_{w}}{\rho_{f}U_{\infty }^{2}},\;\;\text{Nu}=\frac{xq_{w}}{k_{f}\left(T_{f}-T_{\infty }\right)}, \end{array}$$

where *τ*_*w*_ is the skin friction and *q*_*w*_ is the heat flux from the plate which are given by

(11) $$\begin{array}{c} \tau_{w}=\mu_{nf}{\frac{\partial u}{\partial y}|}_{y=0},\;\;q_{w}=-k_{nf}{\frac{\partial T}{\partial y}|}_{y=0}. \end{array}$$

Putting (11) into (10), we obtain

(12) $$\begin{array}{c} \text{R}{\text{e}}_{x}^{1/2}C_{f}=\frac{1}{{\left(1-\varphi \right)}^{2.5}}f^{\prime \prime }\left(0\right),\\ \text{R}{\text{e}}_{x}^{-1/2}\text{Nu}=-\frac{k_{nf}}{k_{f}}\theta^{\prime }\left(0\right). \end{array}$$

The set of (6) and together with the boundary conditions (8) are coupled nonlinear boundary value problems which are solved numerically using a shooting algorithm with a Runge-Kutta Fehlberg integration scheme. This method involves transforming (6) and (8) into a set of initial value problems which contain unknown initial values that need to be determined by guessing, after which a fourth order Runge-Kutta iteration scheme is employed to integrate the set of initial valued problems until the given boundary conditions are satisfied. The entire computation procedure is implemented using a program written and carried out using Maple computer language. From the process of numerical computation, the fluid velocity, the temperature, the skin friction coefficient, and the Nusselt number are proportional to *f*′(*η*), *θ*(*η*), *f*′′(*η*), and *θ*′(*η*), respectively. 

## 3. Results and Discussion 

Physically realistic numerical values were assigned to the pertinent parameters in the system in order to gain an insight into the flow structure with respect to velocity, temperature, skin friction, and Nusselt's number. The results were presented graphically in Figures 2–13, and conclusions are drawn for the flow field. The Prandtl number is kept constant at 6.2 [21]. Ha = 0 corresponds to absence of magnetic field, and *φ* = 0 is regular fluid.

### 3.1. Dimensionless Velocity Profiles

**Figure 2:**
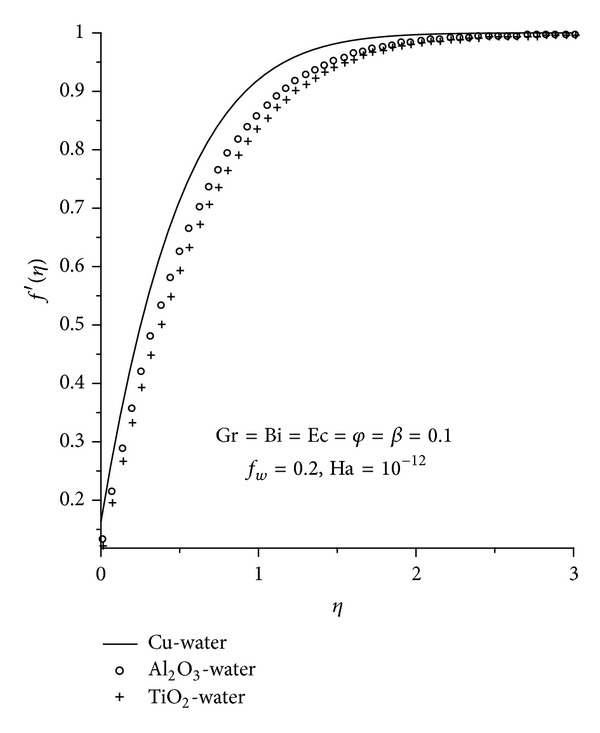
Velocity profiles for different nanofluids.

**Figure 3:**
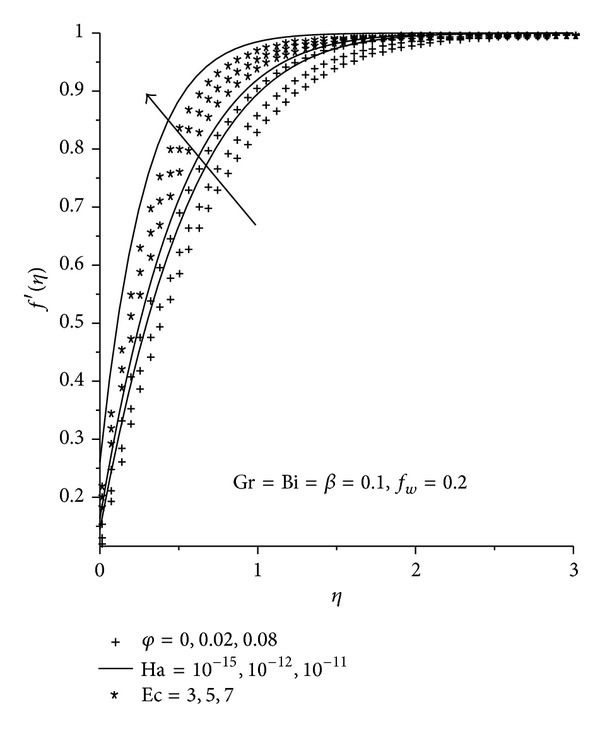
Velocity profiles with increasing Ha, *φ*, and Ec.

**Figure 4:**
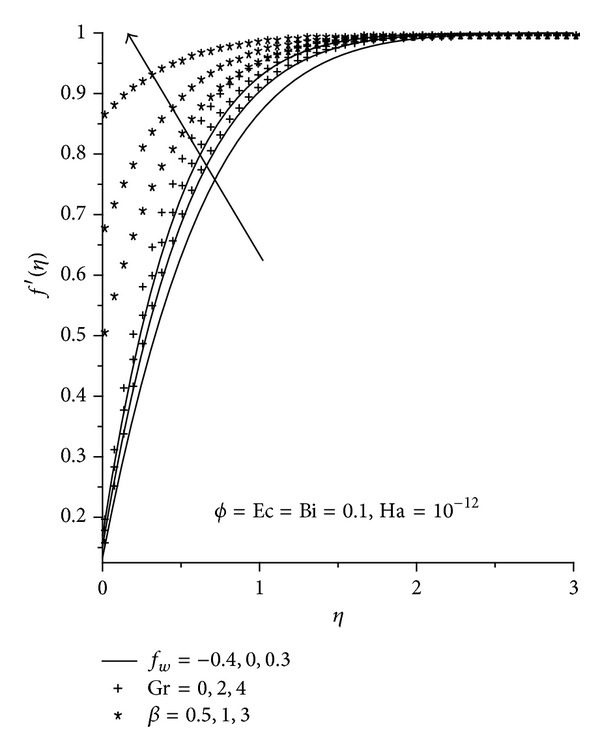
Velocity profiles with increasing Gr, *β*, and *f*_*w*_.

Figures 2–4 illustrate the effects of various thermophysical parameters on the nanofluids velocity profiles. Generally, it is noted that the fluid velocity increases gradually from zero at the plate surface to the free stream prescribed value far away from the plate satisfying the boundary conditions.  Figure 2 shows that the momentum boundary layer thickness for Cu-water nanofluid is smaller than the rest of the nanofluids consequently, Cu-water nanofluid tends to flow closer to the convectively heated plate surface and serves as a better coolant than the other nanofluids. It is observed in Figures 3 and 4 that an increase in the magnetic field intensity (Ha), nanoparticle volume fraction (*φ*), Eckert number (Ec), Grashof number (Gr), and the suction/injection parameter (*f*_*w*_) causes an overshoot of the fluid velocity towards the plate surface hence decreasing both the momentum boundary layer thickness and the fluid velocity. From the physics of the problem, an increase in the magnetic field intensity leads to an increase in the Lorentz force which is a retarding force to the transport phenomena. This retarding force can control the nanofluids velocity which is useful in numerous applications such as magneto hydrodynamic power generation and electromagnetic coating of wires and metal. We also note that the fluid velocity at the plate surface increases with an increase in the slip parameter (*β*). This is in agreement with the fact that higher *β* implies an increase in the lubrication and slipperiness of the surface. 

### 3.2. Dimensionless Temperature Profiles

**Figure 5:**
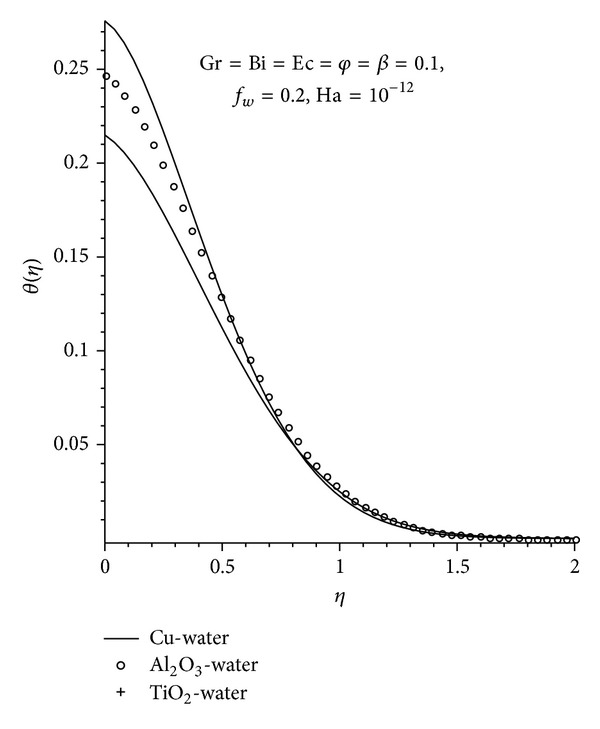
Temperature profiles for different nanofluids.

**Figure 6:**
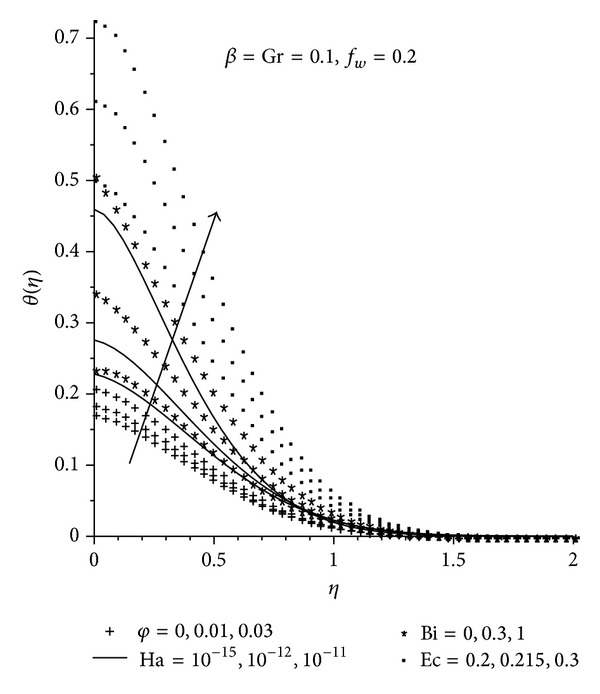
Temperature profiles with increasing *φ*, Ha, Bi, and Ec.

**Figure 7:**
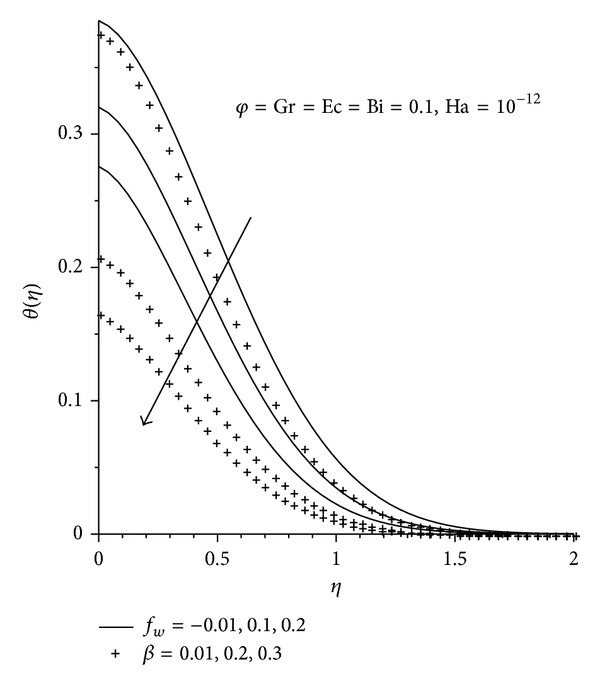
Temperature profiles with increasing *β*, Gr, and *f*_*w*_.

Figures 5–7 show the effects of various parameters on the temperature profile. In general, the maximum fluid temperature is achieved at the plate surface due to the convectional heating but decreases exponentially to zero far away from the plate surface satisfying the free stream conditions. As expected, at the plate surface, Cu-water has the highest temperature and a greater thermal boundary layer thickness than the other two nanofluids, as seen in Figure 5. This is in accordance with the earlier observation, since the Cu-water nanofluid is more likely to absorb more heat from the plate surface owing to its close proximity to the hot surface. It is observed from Figure 6, that increasing Ha, *φ*, Bi, and Ec leads to an increase in both the fluid temperature and the thermal boundary layer thickness. This can be attributed to the additional heating due resistance of fluid flow as a result of the magnetic field, the presence of the nanoparticle, the increased rate at which the heat moves from the hot fluid to the plate and the additional heating as a result of the viscous dissipation.

On the other hand, it is evident that surface slipperiness and suction affect the temperature of the fluid inversely. This is clearly seen from Figure 7, where both temperature and thermal boundary layer decrease as *f*_*w*_ and **β** increase. 

### 3.3. Effects of Parameters Variation on the Skin Friction and Nusselt Number

**Figure 8:**
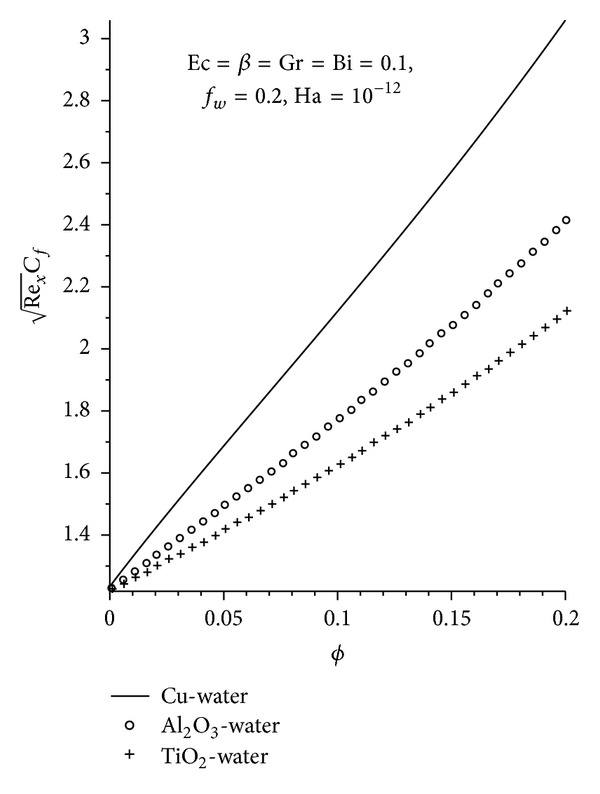
Local skin friction profiles for different nanofluids.

**Figure 9:**
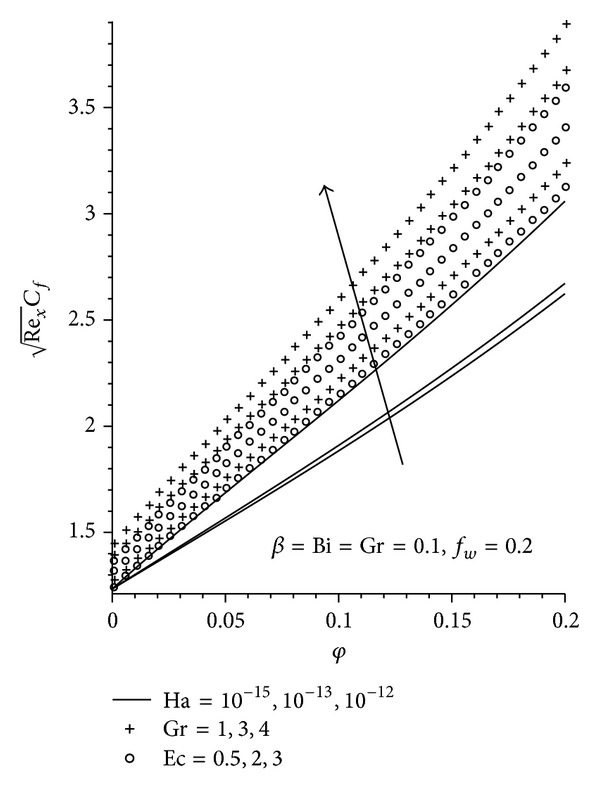
Effects of increasing Gr, Ha, and Ec on local skin friction.

**Figure 10:**
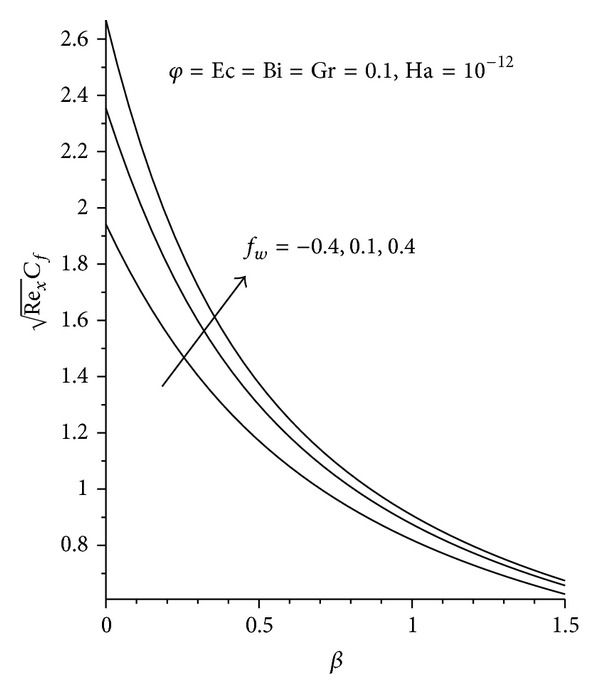
Effects of increasing *β* and *f*_*w*_ on local skin friction.

**Figure 11:**
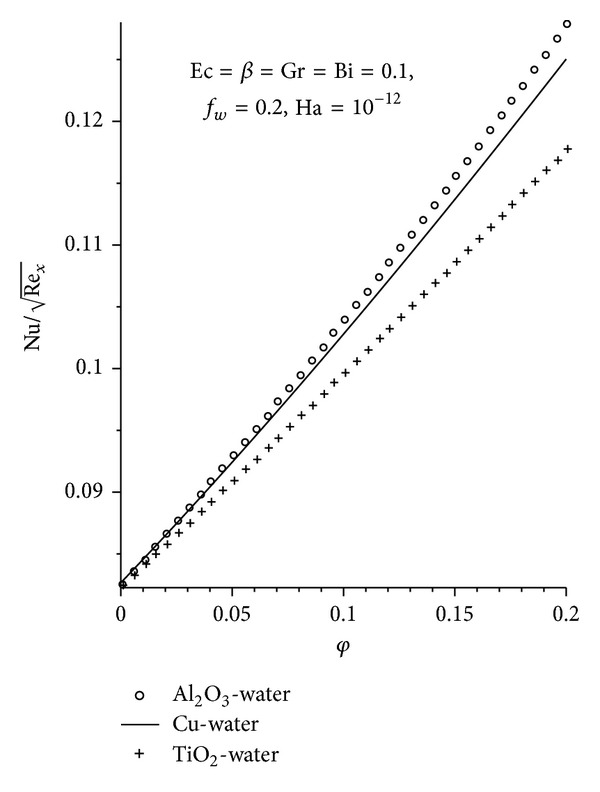
Local Nusselt number for different nanofluids.

**Figure 12:**
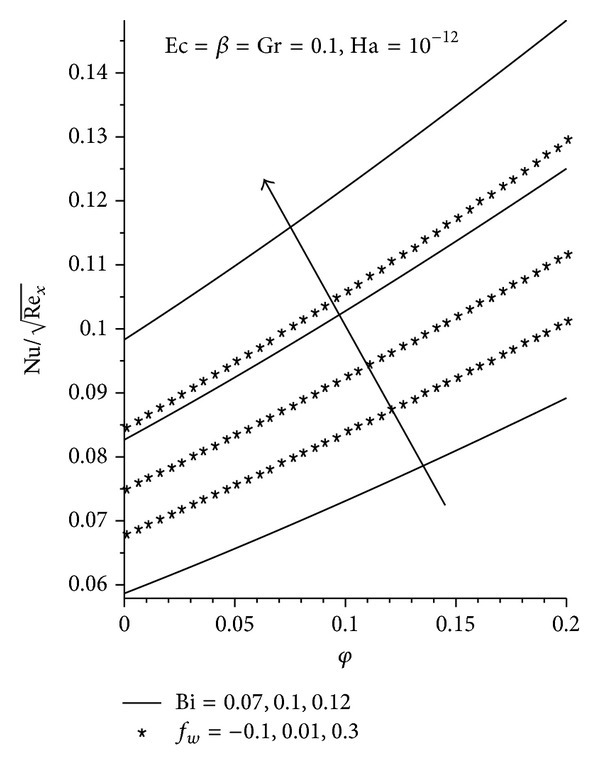
Effects of increasing *φ*, Bi, and Ha on local Nusselt number.

**Figure 13:**
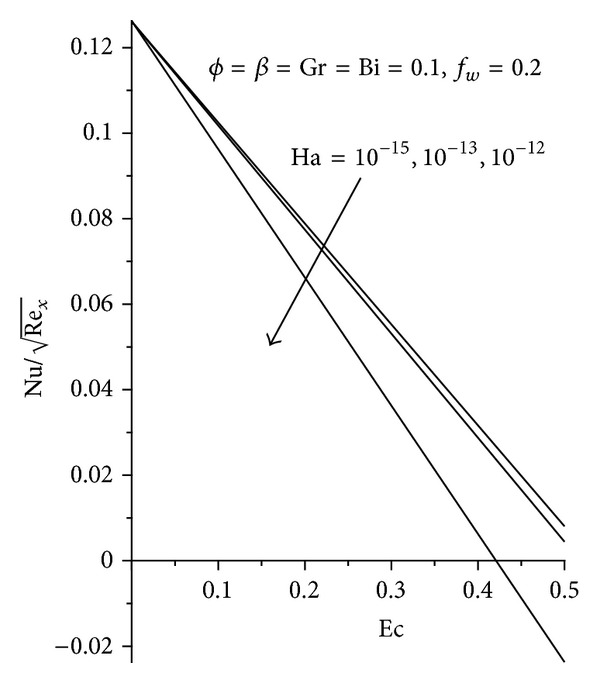
Effects of increasing *φ*, Ha, and Ec on local Nusselt number.

Figures 8–13 demonstrate the effects of the various pertinent parameters at the plate surface for both the skin friction coefficient and the local Nusselt number (rate of heat transfer). The presence of nanoparticle in the convectional fluid leads to an increase in the skin friction, as seen in Figure 8, where increasing the nanoparticle volume fraction increases the skin friction for the three nanoparticles (Cu, Al_2_O_3_, and TiO_2_) used, with Cu-water exhibiting the highest increment. This is as expected, since Cu-water moves closer to the plate surface leading to an elevation in the velocity gradient at the plate surface. As expected, increasing Ha, Gr, Ec, and *f*_*w*_ leads to an increase in the skin friction coefficient, while an increase in **β** reduces the skin friction coefficient as shown in Figures 9 and 10. There is an increase in the rate of heat transfer with an increase in **φ**, Bi, and *f*_*w*_ as seen in Figures 11-12, with Al_2_O_3_ exhibiting the highest increment. The converse is seen with increasing Ha as shown in Figure 13.

## 4. Conclusions

The problem of hydromagnetic boundary layer flow of an incompressible electrically conducting water-based nanofluids past a convectively heated vertical porous plate with Navier slip boundary condition was studied. The governing nonlinear partial differential equations were transformed into a self-similar form and numerically solved using shooting technique with a fourth-order Runge-Kutta-Fehlberg integration scheme, putting into consideration the enhanced electrical conductivity of the convectional base fluid due to the presence of the nanoparticles. Our results showed that the fluid velocity increases, while the local skin friction decreases with the increase in the slip parameter (*β*), but the reverse is observed with the increase in the magnetic field intensity (Ha), nanoparticle volume fraction (**φ**), Eckert number (Ec), Grashof number (Gr), and the suction/injection parameter (*f*_*w*_). Both the temperature and the thermal boundary layer thickness are enhanced by increasing the magnetic field intensity (Ha),nanoparticle volume fraction (**φ**), Eckert number (Ec), and the intensity of Newtonian heating (Bi), while the cooling effect on the convectively heated plate surface is enhanced by increasing the velocity slip (**β**) and suction parameter (*f*_*w*_). 

## Figures and Tables

**Table 1:** Thermophysical properties of water and nanoparticles [23, 24].

Materials	*ρ* (kg/m^3^)	*c*_*p*_ (J/kgK)	*k* (W/mK)	*σ* (S/m)
Pure water	997.1	4179	0.613	5.5 × 10^−6^
Copper (Cu)	8933	385	401	59.6 × 10^6^
Alumina (Al_2_O_3_)	3970	765	40	35 × 10^6^
Titania (TiO_2_)	4250	686.2	8.9538	2.6 × 10^6^
